# Use of spatiotemporal characteristics of ambient PM_2.5_ in rural South India to infer local versus regional contributions^☆^

**DOI:** 10.1016/j.envpol.2018.04.057

**Published:** 2018-08

**Authors:** M. Kishore Kumar, V. Sreekanth, Maëlle Salmon, Cathryn Tonne, Julian D. Marshall

**Affiliations:** a Public Health Foundation of India, New Delhi, India; b Department of Civil and Environmental Engineering, University of Washington, Seattle, WA, United States; c Barcelona Institute for Global Health (ISGlobal), Barcelona, Spain; d Universitat Pompeu Fabra (UPF), Spain; e CIBER Epidemiología y Salud Pública, Spain

**Keywords:** Air pollution, PM_2.5_, Ambient measurements, Sources, India

## Abstract

This study uses spatiotemporal patterns in ambient concentrations to infer the contribution of regional versus local sources. We collected 12 months of monitoring data for outdoor fine particulate matter (PM_2.5_) in rural southern India. Rural India includes more than one-tenth of the global population and annually accounts for around half a million air pollution deaths, yet little is known about the relative contribution of local sources to outdoor air pollution. We measured 1-min averaged outdoor PM_2.5_ concentrations during June 2015–May 2016 in three villages, which varied in population size, socioeconomic status, and type and usage of domestic fuel. The daily geometric-mean PM_2.5_ concentration was ∼30 μg m^−3^ (geometric standard deviation: ∼1.5). Concentrations exceeded the Indian National Ambient Air Quality standards (60 μg m^−3^) during 2–5% of observation days. Average concentrations were ∼25 μg m^−3^ higher during winter than during monsoon and ∼8 μg m^−3^ higher during morning hours than the diurnal average. A moving average subtraction method based on 1-min average PM_2.5_ concentrations indicated that local contributions (e.g., nearby biomass combustion, brick kilns) were greater in the most populated village, and that overall the majority of ambient PM_2.5_ in our study was regional, implying that local air pollution control strategies alone may have limited influence on local ambient concentrations. We compared the relatively new moving average subtraction method against a more established approach. Both methods broadly agree on the relative contribution of local sources across the three sites. The moving average subtraction method has broad applicability across locations.

## Highlights

• Ambient PM_2.5_ levels are monitored in three South Indian villages simultaneously.
• One-minute average PM_2.5_ concentrations were collected over one year.
• PM_2.5_ measured using beta attenuation and photometric methods.
• Compared quantitative, semi-quantitative methods to study spatio-temporal patterns.
• Moving average subtraction method indicated most PM_2.5_ was from regional sources.

## Graphical abstract

**Figure:**
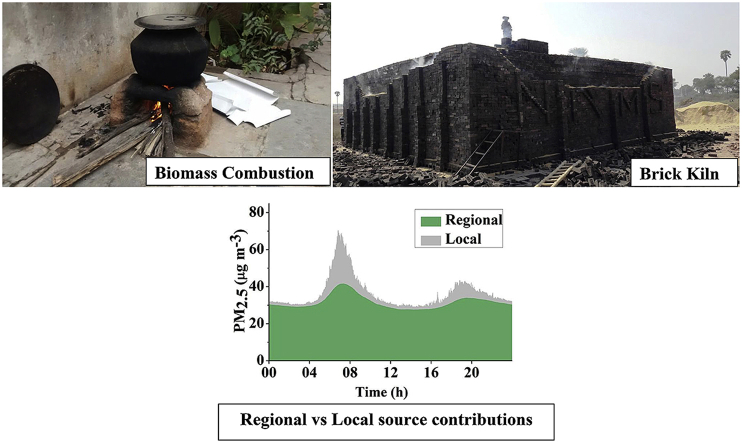

## Teaser

A moving average subtraction method, utilizing 1-min average PM_2.5_ concentrations, indicated contributions from local sources were minor (∼10%); most PM_2.5_ was regional.

## 1 Introduction

Ambient PM_2.5_ air pollution is the third largest risk factor for deaths in India, causing an estimated 1.1 million deaths per year in India, according to the Global Burden of Disease (Cohen et al., 2017). Major sources of PM_2.5_ emissions in rural India include biomass combustion for cooking, lighting, and heating (firewood, charcoal, manure, crop residues), burning of household waste and agricultural residue, traffic, windblown dust, and industry (e.g., brick kilns, rice mills, electricity generation) (Awasthi et al., 2010; Saud et al., 2011; Ministry of Road Transport and Highways, 2012; Rajput et al., 2014; Li et al., 2015; Nirmalkar et al., 2015; Aung et al., 2016; Pant et al., 2016; Vreeland et al., 2016). India has ∼0.75 billion rural biomass users, emitting more than 2 million tons of PM_2.5_ annually (Census, 2011; Chakraborty et al., 2014; Cofala et al., 2015).

The existing literature on outdoor air pollution in rural India primarily focuses on North India (Awasthi et al., 2010; Kulshrestha et al., 2009; Massey et al., 2013; Nirmalkar et al., 2015; Pachauri et al., 2013; Rajput et al., 2014; Rastogi et al., 2016; Shandilya et al., 2007); few peer-reviewed studies exist for the South (see literature review, below). Important South/North differences in India include climate and meteorology, crops and vegetation, types of local sources (e.g., types of biomass used for cooking, technology of brick kilns), culture, and population density (Maithel et al., 2012; Guttikunda et al., 2014; Pant et al., 2016).

Information regarding emission sources is important for scientific understanding of air pollution and to inform policymakers. Few studies have explored how temporal patterns in concentrations can be used to estimate the relative contribution of local versus regional sources. One study, by Watson and Chow (2001), demonstrated that temporal decomposition of real-time concentrations can shed light on the proportion of concentrations that are attributable to local sources. To our knowledge, this approach was subsequently employed by other researchers only twice (Apte et al., 2011; Both et al., 2011).

In this study, we report results from 12 months of 1-min ambient PM_2.5_ measurements at three locations in rural South India. Our objectives were to (1) characterize spatiotemporal patterns, and (2) based on those patterns, quantify local and regional source contributions using multiple analytical approaches. Our study provides new knowledge by advancing and comparing methods for understanding likely emission sources based on spatial and temporal patterns of PM_2.5_ air pollution. Approaches developed here could usefully be applied to other time periods or locations. As a secondary contribution to new knowledge, we provide measurements for a region (rural southern India) with a substantial health burden from air pollution, yet whose air pollution is poorly studied. Monitoring presented in this paper is part of the Cardiovascular Health effects of Air pollution in Telangana, India (CHAI) project, an epidemiology study investigating drivers of population exposure to particles and their health effects (Tonne et al., 2017).

## 2 Materials and methods

**Fig. 1:**
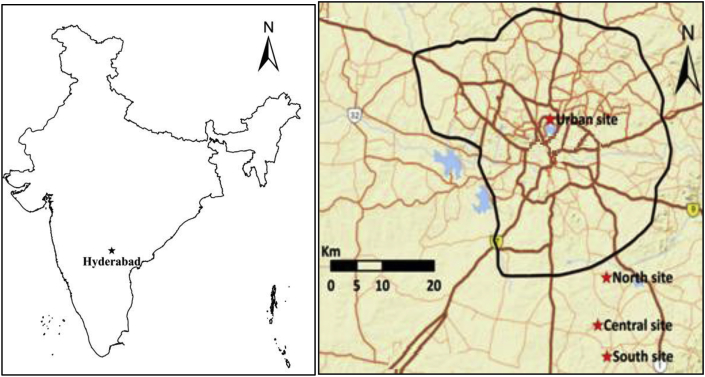
Monitor locations.

**Monitor locations.** We monitored real-time ambient PM_2.5_ mass concentrations in three villages in Ranga Reddy district, Telangana; sites are 22–35 km southeast of Hyderabad (Fig. 1). We selected three locations to cover varying population size, village-level socioeconomic status and primary fuel-type for household activities, while also meeting logistical requirements (e.g., accessible, secure locations for instruments; reliable access to electricity). Sites (Table 1) were selected that were not immediately next to a major road or other source of pollution. The Central monitor was 9 km (6 km) from the North (South) monitor.

**Table 1:** Description of monitoring sites.

Site (Village name; monitoring station coordinates)	Village population^a^	Primary domestic fuel^a^		Instruments employed			
		LPG	Biomass	eBAM	DustTrak	RH sensor	Weather station
North site (Sahebguda; 17°12′5.85″N, 78°34′19.47″E)	100	✓		✓	✓	✓	✓
Central site (Timmapur; 17°7′45.08″N, 78°33′25.42″E)	3000	✓	✓		✓	✓	✓
South site (Gumadavalli Tanda; 17°4′54.87″N, 78°34′22.91″E)	400		✓	✓	✓	✓	✓

a Basis: questionnaire survey in the respective village panchayats.

**PM**_**2.5**_ **measurements.** DustTrak aerosol monitors (Model 8530, TSI Inc., Shoreview, MN) measured real-time PM_2.5_ concentrations. DustTraks use a laser photometer to estimate PM_2.5_ mass concentrations based on 90° light scattering (TSI, 2014a). Sheath air within the instrument keeps the optics clean for improved reliability and low maintenance. We employed manufacturer-supplied PM_2.5_ inlet nozzles and impactors, with 1 min sampling interval and 3 Lpm flow rate (corresponding to 2.5 μm cut-point) (TSI, 2014b). We corrected the DustTrak-recorded real time PM_2.5_ concentrations for real-time relative humidity (Both et al., 2011) and daily average local aerosol properties (Ramachandran et al., 2003; Wallace et al., 2011; Apte et al., 2011).

eBAMs (Model 9800, MetOne, Grants Pass, OR), which work by beta attenuation method for the measurement of PM_2.5_ mass concentration (Cheng et al., 2008; Cisneros et al., 2014; Schweizer et al., 2016) eBAMs were deployed in North and South sites. eBAMs are relatively mobile instruments and intended for temporary deployment, although have shown good correlation (R^2^ = 0.9 for daily mean) with non-mobile federal equivalent method BAM instruments (Schweizer et al., 2016). We employed manufacturer-supplied inlet air heating to avoid humidity-related errors (MetOne, 2011) and manufacturer-supplied PM_2.5_ cyclones, with 15 min sampling frequency and 16.7 Lpm flow rate (corresponding to 2.5 μm cut point).

**Meteorological measurements.** 1-min averaged relative humidity (RH) was measured using LabJack (Model: Digit-TLH, LabJack, Lakewood, CO) and Hobo (Model RH481, Onset, Bourne, MA) monitors. Weather stations (Model: PWS1000 TB, Zephyr Instruments, East Granby, CT) with anemometer, wind wane, temperature sensor and data logger recorded at 30-min frequency.

**Maintenance.** Instruments were inspected, cleaned, and calibrated approximately weekly to check for instrumental errors. Inspection included zero-check (and, for the DustTrak, recalibration) and flow-check (digital flow meter; model: Bios Defender 510, Mesa Labs, Lakewood, CO). The eBAM inlet and cyclone were cleaned monthly per manufacturer's requirements (MetOne, 2011).

**RH Correction.** Ambient conditions above 60% RH favor hygroscopic growth of particles, leading to overestimation of PM_2.5_ measurements by the DustTrak (Apte et al., 2011; Ramachandran et al., 2003). For PM_2.5_ concentration measurements sampled when RH >60%, we corrected the DusTrak readings using equations (1), (2)), developed by Chakrabarti et al. (2004). Ambient conditions above 95% RH may result in large distortions in DustTrak data and were excluded from analysis. Both et al. (2011) documented the importance of real-time (rather than time-average) RH-correction.

(1) $$\;CF=1+0.25\frac{RH^{2}}{\left(1-RH\right)}\;$$

(2) $${\text{PM}}_{2.5\;\text{RH}-\text{Corrected}}=\frac{PM_{2.5}}{CF}\;$$

**Reference correction.** Aerosol optical properties at the measurement site may differ from those used during factory calibration (Chung et al., 2001; Yanosky et al., 2002; Wallace et al., 2011; McNamara et al., 2011). To obtain correction factors, we collocated all DustTraks and eBAMs in North and South sites during various periods of the monitoring campaign. RH-corrected 24 h average DustTrak values were compared and regressed against the respective 24 h average eBAM PM_2.5_ measurements; that regression yields the DustTrak calibration curve.

**Sampling design.** The monitoring campaign was designed to capture rural PM_2.5_ levels during all seasons (monsoon [June–September], post monsoon [October–November], winter [December–February], summer [March–May]). PM_2.5_ measurements started in June 2015 and continued until the end of May 2016 in North and Central sites. At the South site, monitoring was carried out until the end of April 2016 because of limitations in instrument availability. Analyses are based on (RH- and eBAM-corrected) DustTrak PM_2.5_ data because of the DustTrak's higher temporal resolution than the eBAM. eBAM-measured PM_2.5_ was also used to impute data during maintenance and non-operational periods of DustTraks at North and South sites.

### 2.1 Data analysis

**Characterizing spatiotemporal patterns in PM**_**2.5**_. We compared 24 h average concentrations at the three sites according to international and national benchmarks as well as against a nearby urban site. We used the following classification for 24 h values: “low” (below the corresponding World Health Organization (WHO) guideline of 25 μg m^−3^), “medium” (above 25 μg m^−3^ but below the Indian National Ambient Air Quality (NAAQ) standard of 60 μg m^−3^), and “high” (above 60 μg m^−3^). We define a PM_2.5_ episode as any hour with average concentration greater than the Indian 24 h NAAQ standard (60 μg m^−3^) (Wang et al., 2015). Hourly PM_2.5_ concentrations measured at a nearby urban site were available from the U.S. Consulate in central Hyderabad.

**Local and regional scale contributions.** To investigate contributions of local and regional sources, we applied a moving average subtraction method similar to that developed by Watson and Chow (2001) and employed by Both et al. (2011) and Apte et al. (2011). Briefly, 1 min averaged PM_2.5_ concentrations were smoothed at multiple timescales (6 h, 3 h, 1.5 h, 45 min, and 15 min), always selecting the lowest values. Short-duration concentration pulses are hypothesized as attributable to local sources (less than ∼0.5 km). Concentrations after removing the short-term spikes are interpreted as regional plus long-range contributions (greater than ∼0.5 km). As a sensitivity analysis, we also used an alternate underwriting function (see SI 1.1).

**Atmospheric transport analysis.** We defined atmospheric conditions as “stagnation”, “ventilation”, and “recirculation” based on Allwine and Whiteman (1994). Further, we calculated average daily critical transport indices for ventilation using methods from Chithra and Nagendra (2014). See SI 1.2 for a description of these methods.

## 3 Results

### 3.1 Photometer corrections

The prevalent climate of our study region is semi-arid. RH was low (<60%) most (∼55%) hours, resulting in hourly correction factors (CF) for DustTrak measurements that were unity 55% of hours, and averaged ∼1.24 overall (Figure SI.2.1). Non-unity CFs (45% of hours) are generally during late nights/early mornings (21:00–07:00). Linear regression appeared to provide a reliable calibration for correcting the (RH-corrected) DustTrak to the eBAM measurements (R^2^ = 0.90; Figure SI.2.2). The correction factor derived here is consistent with previous studies (e.g., Yanosky et al., 2002; Branis and Hovorka, 2005; Both et al., 2013). All DustTrak values reported below are RH-and eBAM-corrected.

### 3.2 Daily-averages

**Fig. 2:**
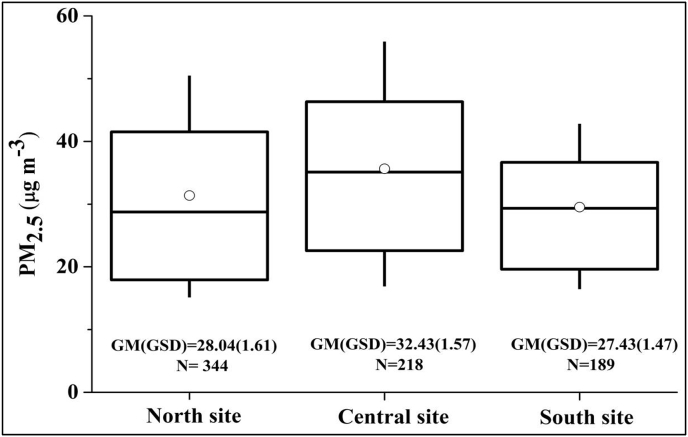
Distribution of 24 h average PM_2.5_ concentrations. GM: geometric mean, GSD: geometric standard deviation. Here and elsewhere, box plots represent the following statistical parameters: median (central horizontal line), mean (circle inside the box), 25th and 75^th^percentiles (box), and 10th and 90^th^ percentile (whiskers). Sample size (e.g., N = 344 for North site) indicates number of days of data (24 h averages) used to make the boxplot.

24 h average PM_2.5_ mass concentrations were approximately lognormally distributed (Figure SI.2.3). Distributions of all data collected during the monitoring period are presented by site in Fig. 2. Based on daily-average PM_2.5_ concentrations among the three sites, PM_2.5_ pollution was “low” 29–42% of days, “medium” 54–66% of days, and “high” 2–5% of days. The total hours of PM_2.5_ episodes were 615 (7%), North; 485 (9%), Central; and 179 (4%), South.

### 3.3 Temporal variation in PM_2.5_ at rural sites

**Fig. 3:**
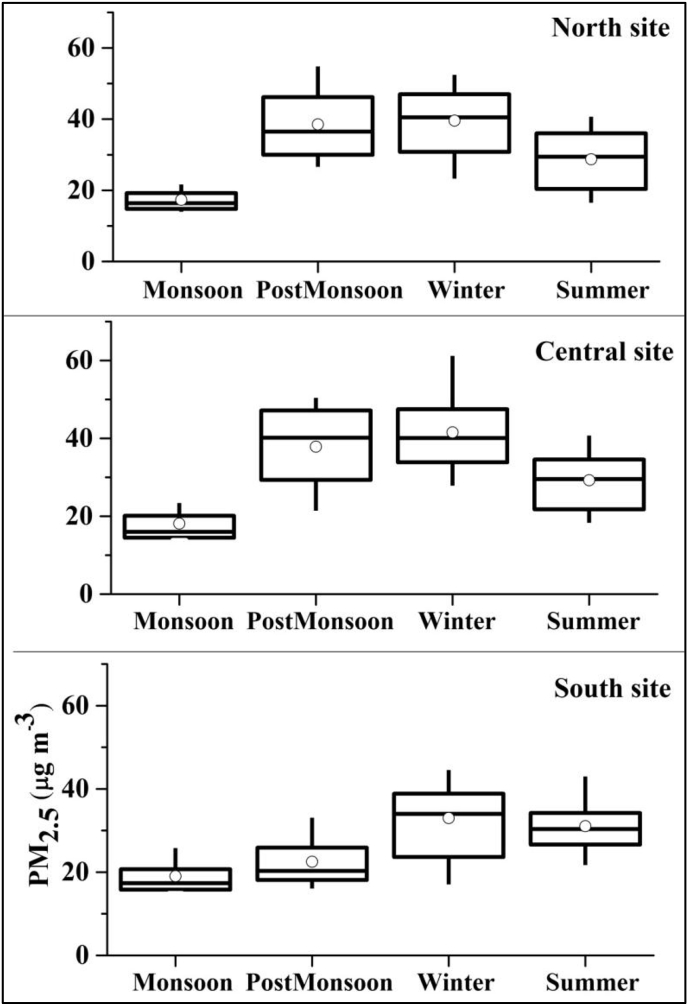
24 h average PM_2.5_ concentrations by site and season using all available data (sample sizes given in Fig. 2).

Concentrations were highest during post monsoon and winter seasons, followed by summer. Concentrations were lowest during the monsoon season (Fig. 3, SI.2.4). During winter, daily-average concentrations exceeded the WHO guideline 76–98% of days and PM_2.5_ episodes existed 7–19% of hours. During monsoon, the WHO guideline was exceeded 4–13% days and 0–2% of total hours were PM_2.5_ episodes. Concentrations during weekdays and weekend days were similar (Table SI.2.1).

### 3.4 Spatial variation at rural sites

**Fig. 4:**
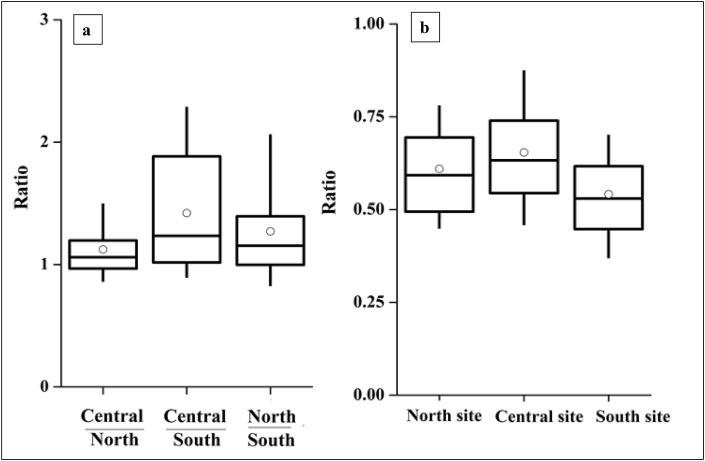
Ratios of 24 h average PM_2.5_ mass concentrations: (a) ratios among the three sites and (b) rural-to-urban ratios.

To explore spatial variability of PM_2.5_ concentrations, we restricted analyses to the 139 days with simultaneous data for all three sites (Figure SI.2.5). Concentration differences among the three sites were statistically significant (null hypothesis: μ_North_ = μ_Central_ = μ_South_ rejected, p < 0.05). Median (25^th^-75th percentile) PM_2.5_ concentrations (units: μg m^−3^) among days in common were 37 (22–46), North; 40 (26–48), Central; 27 (19–37), South. Concentration ratios for pair-wise comparison of the sites (Fig. 4(a)) support the finding that the three sites are similarly polluted, with concentrations slightly lower at the South site, although spatial variability differed by season (Table SI.2.2). Seasonal variation was greater for Central and North compared to the South site (Figure SI.2.6).

**Fig. 5:**
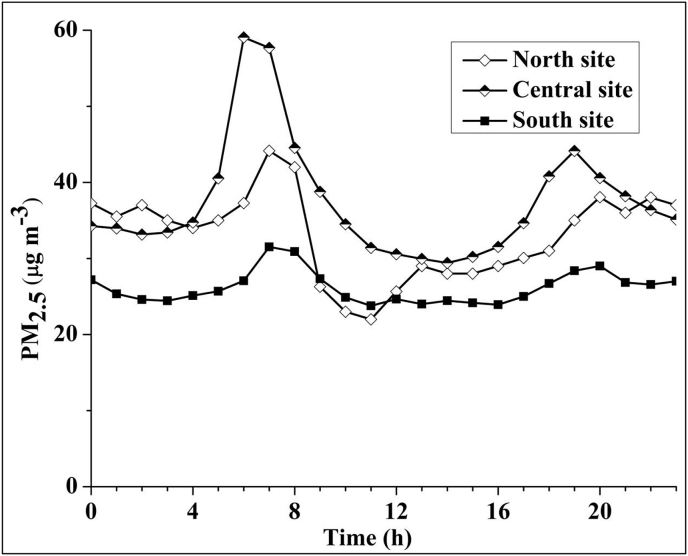
Median PM_2.5_ concentration by time of day based on the common days' data among the three sites.

Spatial variability differed by hour of day (Fig. 5). During morning peak hours (05:00–09:00), concentrations at the Central site were 30% and 68% higher than North and South sites, respectively (spatial coefficient of variability [CV]: 28%). During evening peak hours (17:00–19:00), Central site concentrations were 25 and 49% higher than North and South sites, respectively (CV: 20%). During night (20:00–04:00), Central and North site concentrations were higher than South site (CV: 15%). However, during afternoons, the concentrations were generally similar among the three sites (CV: <10%).

**Fig. 6:**
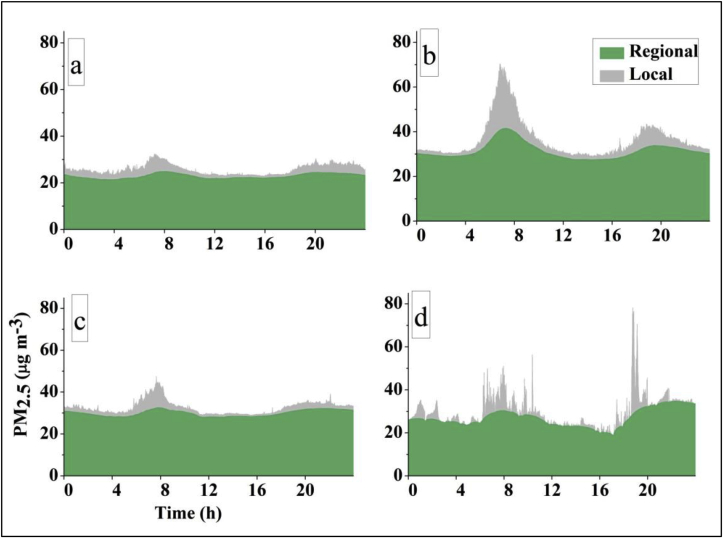
Median PM_2.5_ concentration by local and regional scale contributions, by time of day at (a) North, (b) Central and (c) South sites, based on moving-average subtraction. (d) Example of local and regional concentrations for one 24 h period (Jan 18, 2016; Central site).

Using 1 min averaged concentrations, we computed the ratio of the 95th percentile for certain times to the daily median for that day; as Apte et al. (2011) report, this metric informs the strength of local source emissions during various intervals of day. The 95th percentile of 1 min averaged concentrations during morning peak hours (05:00–09:00) exceeded the daily median by 3.2 at the Central site, which is consistent with strong local emission sources (Figure SI.2.7). Fig. 6 and Table SI 2.3, too, reveal impacts of local emission sources at the Central site.

### 3.5 Comparison with urban PM_2.5_ levels

Daily-average PM_2.5_ concentrations were ∼35–47% lower in the three rural sites than at the nearby urban monitor (U.S. Consulate, Hyderabad) (Fig. 4(b)). The rural-urban gap was reduced slightly, to ∼22–46%, during winter, and increased slightly, to ∼38–50%, during monsoon (Figure SI.2.8). Daily-average concentrations were lower in rural than in urban areas 99% of the time; the reverse pattern (overall, 1% of the time) occurred only during winter.

### 3.6 Local and regional source contribution

As mentioned above, Watson and Chow (2001) showed that temporal decomposition of real-time concentrations can shed light on the relative importance of local versus regional sources. The temporal decomposition approach is best suited for relative, rather than absolute, comparisons across sites or times. To our knowledge, only two other articles used the moving average subtraction method: Both et al. (2011) and Apte et al. (2011) applied and extended that approach for India-specific data in urban areas (Bangalore and Delhi, respectively). Our analyses, employing similar approaches, reveal the following. On average, local sources contributed ∼8–12% among the three rural sites. Local contributions were highest (16–25%) during morning peak hours (Table SI.2.3). At the Central site, average PM_2.5_ concentrations from local sources were ∼1.7 × higher than the other two sites, contributing ∼25% during morning, 17% during evening peak hours (Fig. 6). During afternoon periods, contributions from local sources were minor (5–8%) at all three sites. Results from the sensitivity analysis using an alternative underwriting function (see SI) reveal similar patterns (Figure SI.2.9 and Table SI.2.4), although the alternative approach overall apportioned a somewhat smaller proportion of concentrations to local sources.

### 3.7 Meteorological effects

Following the approach of Allwine and Whiteman (1994), we find that 61–64% of days include stagnation, 41–49% of days include recirculation, and 6–16% of days include ventilation. (Those categories are not mutually exclusive when used to categorize meteorological conditions for each day; 29–34% of days obtained more than one label; see Table SI.2.5). As expected, PM_2.5_ concentrations were higher during stagnation periods and lower during ventilation periods (Table 2). During winters, stagnation days were prevalent (35–55% of days); concentrations were higher during winter than other times. During monsoon, there were relatively more ventilation periods (50–55% of days) and fewer stagnation periods (2–20% of days) (Table SI.2.6); concentrations were lower during monsoon than at other times.

**Table 2:** Percentage of days with stagnation, recirculation and ventilation conditions; average concentrations and estimated local source contribution during these periods.

	Stagnation			Recirculation			Ventilation		
	% of days of occurrences	Average concentration (μgm-3)	Percentage of local source contribution	% of days of occurrences	Average concentration (μgm-3)	Percentage of local source contribution	% of days of occurrences	Average concentration (μgm-3)	Percentage of local source contribution
North site	64	34	15	44	34	10	16	24	9
Central site	63	38	16	41	32	13	8	25	11
South site	61	30	10	49	30	6	6	27	6

Calm conditions resulted in higher than average PM_2.5_ concentrations and more frequent PM_2.5_ episodes (Table SI.2.7). Mapping of known local emission sources (Figure SI. 2.10), and analysis of concentration by wind direction (Figures SI. 2.11-2.13), are consistent with local sources such as brick kilns and rice mills being important local sources; PM_2.5_ levels were higher when monitoring sites were downwind of those sources (Table SI.2.8).

To quantify the local source contribution under various meteorological conditions, we applied the moving average subtraction method separately for each of the three meteorological classes (stagnation, recirculation, ventilation). Results indicated that the local source contribution is highest (10–16%) during stagnation, versus 6–13% during recirculation and 6–11% during ventilation (see Table 2). Those relative patterns are consistent with expectations and suggest internal consistency among the methods.

## 4 Discussion

We employed a computationally effective analytical approach, the moving average subtraction method, which can inform the relative contribution of local sources to ambient concentrations based on temporal patterns. Results from the moving average subtraction method were broadly consistent with the atmospheric transport analysis, both of which investigate contributions of nearby local sources (e.g., biomass and agricultural crop burning, and rural industries such as brick kilns and rice mills). We observed greater spatial variability in PM_2.5_ concentrations than reported in the existing literature (Dey et al., 2012). However, the majority of PM_2.5_ was regional. Because the local sources observed in our study location are relatively common, we hypothesize that our findings regarding local source contributions to PM_2.5_ likely apply broadly to rural South India. The moving average subtraction method can be applied to just one or many monitoring locations; here, we applied it to three monitor-locations in our study area.

These kinds of results are unlikely to be achieved from routine monitoring or source apportionment techniques; they point to unique advantages of the moving average subtraction method developed by Watson and Chow. A combination of a high-resolution (spatiotemporal) emission inventory and air quality models could potentially yield similar findings to what is presented here, but would be computationally intensive and would depend on data availability (e.g., the high-resolution local emission inventory). When we compared results from the moving average subtraction method against those from a more established approach (classification by atmospheric transport conditions), we found substantial agreement.

The moving average subtraction method and the underwriting function attributed comparatively higher percentages of local contribution at the central site than at the North and South sites. Thus, our spatiotemporal analyses identified the largest contribution of local sources to measured PM_2.5_ at the central site, as expected given its larger population density. Nonetheless, regional source contribution to PM_2.5_ was around 9 times more than that of local sources across all sites. Results from both techniques broadly agree regarding the greater influence of local sources at the central site and that most PM_2.5_ is attributable to regional sources.

Higher winter concentrations likely reflect increased emissions from domestic biomass combustion, and commencement of brick kiln operation and agricultural crop residue burning. In addition, shallow mixing heights and stagnant conditions result in lower ventilation coefficients (Sujatha et al., 2016), which reduces dilution rates. We observe wintertime concentrations higher by ∼25 μg m^−3^ than monsoon levels at all three sites. During summer, higher surface temperatures and mixing heights favor atmospheric convection, which promotes larger dispersion and more rapid mixing (dilution) of PM_2.5_ (Sujatha et al., 2016; Table SI.2.9). In addition, local survey results indicate reduced consumption of biomass for household activities such as water heating for bathing during summer, which could have reduced local PM_2.5_ emissions in summer relative to winter. During monsoon, concentrations were low, which reflects dilution rates and increased particle scavenging (wet deposition) (Dumka et al., 2013). Furthermore, brick kiln operations and crop residue burning terminate, reducing local PM_2.5_ emissions, during monsoon. Peak-hour concentrations, especially during morning peak, likely reflect comparatively stagnant air as well as high household emissions. Individuals may spend substantial portions of time in ambient environments during these periods (travel to workplaces and schools, work in nearby agricultural fields); therefore, the high concentrations during this period may make a relatively large contribution to total daily exposure to PM_2.5_.

Concentrations at these rural Indian sites exceeded typical concentrations measured in rural areas of the US and Europe but were lower than those measured in rural North India. Typical PM_2.5_ concentrations reported for rural sites in developed countries such as the US, Canada, and Europe are ∼ 6–15 μg m^−3^ (Cheng et al., 2000; Kundu and Stone, 2014; Schwarz et al., 2016), or about 50–80% lower than results here. On the other hand, PM_2.5_ concentrations reported for North Indian villages are ∼100–150 μg m^−3^ (Kulshrestha et al., 2009; Massey et al., 2009; Dey et al., 2012), or 3–5 × greater than those in the present study. The higher concentrations in rural North India reflect differences in emissions and dilution. For example, the Northern locations, which may be colder during winter than locations studied in this work, may involve greater usage of biomass and coal for cooking and heating during winters (Guttikunda et al., 2014). Most (∼65%) of brick kilns in India are located in the North (Maithel et al., 2012). Atmospheric dilution rates in general are lower for the North than for the South (Attri, 2008). A literature review for recent studies (published during 2007–2017) on ambient PM_2.5_ air pollution in rural India is summarized in Table 3. Reported rural concentrations generally are higher for North India (10 out of 13 papers) than South India (2 out of 13 papers: one prior article, plus the present article).

**Table 3:**
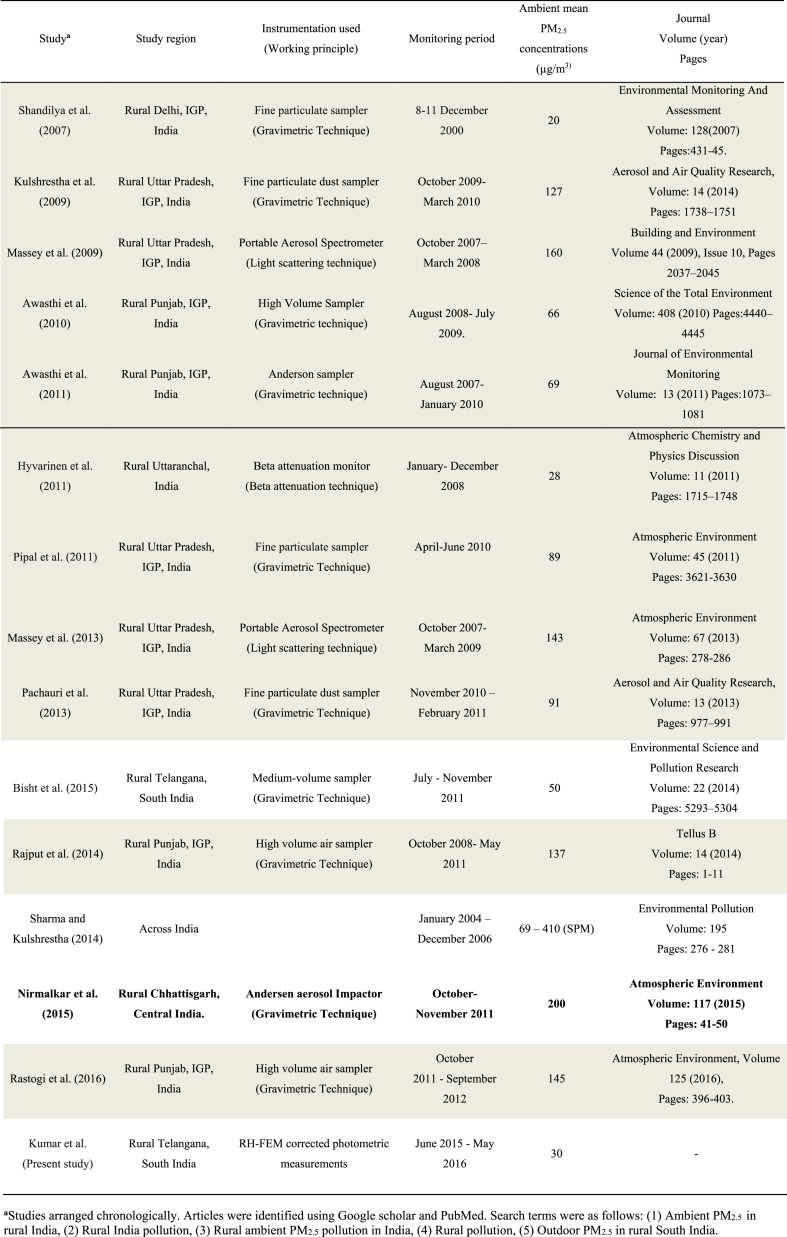
Studies during 2007–2017 reporting ambient PM_2.5_ measurements in rural India: Indo Gangetic Plane (IGP) locations (grey), Central India locations (bold) and South India locations (no highlight) (Awasthi et al., 2011, Bisht et al., 2015, Hyvärinen et al., 2011, Pipal et al., 2011, Sharma and Kulshrestha, 2014).

Ambient air quality monitoring in India has largely focused on urban areas, with limited monitoring in rural sites (Dey et al., 2012; Balakrishnan et al., 2014). India's Central Pollution Control Board (CPCB) operates more than 683 monitoring stations in 300 cities and towns across India (CPCB, 2017). However, most Indians live in rural India (∼70%, or ∼0.8 billion people), and most of them (∼90%) are biomass users (Census, 2011; Chakraborty et al., 2014). Further, Cofala et al. (2015) estimated that biomass burning contributes more than 50% of total Indian PM_2.5_ emissions. Hence, there is a need for more monitoring at rural Indian sites, to generate data needed to understand sources, and quantify population exposures and health impacts (Dey et al., 2012; Chafe et al., 2014; Cofala et al., 2015; Sagar et al., 2016). Future studies on chemical and biological composition of ambient PM_2.5_ would shed further light on sources of pollution, providing additional information for air pollution mitigation.

To summarize, the new findings of the study include (1) advancing quantitative and semi-quantitative methods for investigating spatial and temporal patterns in air pollution, including inter-comparison of multiple such methods, and (2) estimating local and regional contributions to observed ambient PM_2.5_ in rural India.
