# Supplementary material for: Use of spatiotemporal characteristics of ambient PM2.5 in rural South India to infer local versus regional contributions
Source: Environ Pollut. 2018 Aug;239:803–11. doi: 10.1016/j.envpol.2018.04.057 (PMC5980999; doi:10.1016/j.envpol.2018.04.057)
Supplement: Supplementary information [file mmc1.pdf]

Supplementary information

Use of spatiotemporal characteristics of ambient PM2.5 in rural South India to infer local versus regional contributions

M. Kishore Kumar, V. Sreekanth, Maëlle Salmon, CathrynTonne,
Julian D. Marshall

Contents

SI.1 methods
SI. 1.1 Alternate under-writing function
SI. 1.2 Estimation of atmospheric transport and diffusion

SI. 2 Results
Figure SI. 2.1. Real time RH correction
Figure SI. 2.2. eBAM-DustTrak collocation
Figure SI. 2.3.Distributions of $PM_{2.5}$ concentrations measured at three ambient sites
Figure SI. 2.4.Week wise PM $_{2.5}$ at the three rural sites
Figure SI. 2.5.Data Availability
Figure SI. 2.6. Season wise PM $_{2.5}$ variations at the three sites based on common days data
Figure SI. 2.7.Box plots of skew variations at three rural sites
Figure SI. 2.8.Season wise urban-rural PM $_{2.5}$ variations
Figure SI. 2.9.Local and regional scale contributions using alternate underwriting function
Figure SI. 2.10.Locations of brick kilns and rice mills around three rural sites
Figure SI. 2.11. Season wise wind roses for North site
Figure SI. 2.12. Season wise wind roses for Central site
Figure SI. 2.13. Season wise wind roses for South site
Table SI. 2.1. $PM_{2.5}$ concentrations during weekdays and weekends
Table SI. 2.2. Season wise ratios of $PM_{2.5}$ levels at the three ambient sites
Table SI. 2.3. Local source contributions based on moving average subtraction method
Table SI. 2.4. Local source contributions based on alternate underwriting function
Table SI. 2.5. Percentage of days with stagnation, recirculation, ventilation, and stagnation-recirculation events
Table SI. 2.6.Percentage of stagnation, recirculation and ventilation events during various seasons
Table SI. 2.7.Hourly median $PM_{2.5}$ concentrations ($\mu g\ m^{-3}$) during various wind speeds.
Table SI. 2.8.Hourly median $PM_{2.5}$ concentrations ($\mu g\ m^{-3}$) during various wind directions
Table SI.2.9.Average and standard deviations of monthly temperature and relative humidity (RH) over the study area

SI.1 methods

SI. 1.1 Alternate under-writing function

We used an alternate under writing function to validate the results from the moving average subtraction method. In the past, few studies reported that the moving average subtraction method often underestimates the proportion of concentrations attributable to local emissions and over estimates the strength of regional sources (Both et al., 2011). In the present study, we employed an alternative underwriting function that selects, for each time point, the second lowest value from a 360 min moving window.

SI. 1.2 Estimation of atmospheric transport and diffusion

We estimated atmospheric conditions by means of stagnation, ventilation, and recirculation analysis proposed by Allwine and Whiteman (1994). During stagnation,winds are slow or stagnant altogether, resulting in accumulation of pollutants in the vicinity of a source. During recirculation,pollutants are initially transported from source but later return; this pattern mayresult a pollution episode. During ventilation,polluted air is carried away by fresh air.

We calculated the representative integral quantities for stagnation and recirculation using measured wind speed (U_i) and direction (D_i). For a given wind speed and direction, the wind components were expressed as follows:

$$n_i = U_i cos(D_i - 180) \quad (1)$$

$$e_i = U_i sin(D_i - 180) \quad (2)$$

Where i =1, . . . ,N,n_i and e_i represent North-South and east-west component respectively for each discrete data point i.

The North-South transport distance (X_i) and east-west transport distance (Y_i) for an averaging time interval T were estimated using equation (3) and (4), respectively.

$$X_i = T \sum_{j=i}^{i+p} n_j \quad (3)$$

$$Y_i = T \sum_{j=i}^{i+p} e_j \quad (4)$$

where, i =1, . . . ,N-p; $p = \tau/T - 1$; τ = Transport time.

The resultant transport distance (L_i) was computed as

$$L_i = \sqrt{X_i^2 + Y_i^2} \quad (5)$$

The wind run (S_i) and recirculation factor (R_i) were computed as

$$S_i = T \sum_{j=i}^{i+p} U_j \quad (6)$$

$$R_i = 1 - \frac{L_i}{S_i} \quad (7)$$

If $S_i \leq S_c$, asite is prone to stagnation.

If $R_i \geq R_c$, asite is prone to recirculation.

If $R_i \leq R_{cv}$and $S_i \geq S_{cv}$, a site is prone to ventilation.

Here, S_c and R_c are average daily critical transport indices (CTIs) for stagnation and recirculation, respectively. The CTI parametersare estimated as the average wind run and average recirculation factor during the monitoring period, respectively. S_{cv} and R_{cv} are the CTIs for ventilation and are computed as 75th percentile of S_i and 25th percentile of R_i, respectively (Chithra et al., 2014). The transport labels ("stagnation" and "recirculation") are not mutually exclusive, that is, a day might receive more than one label.

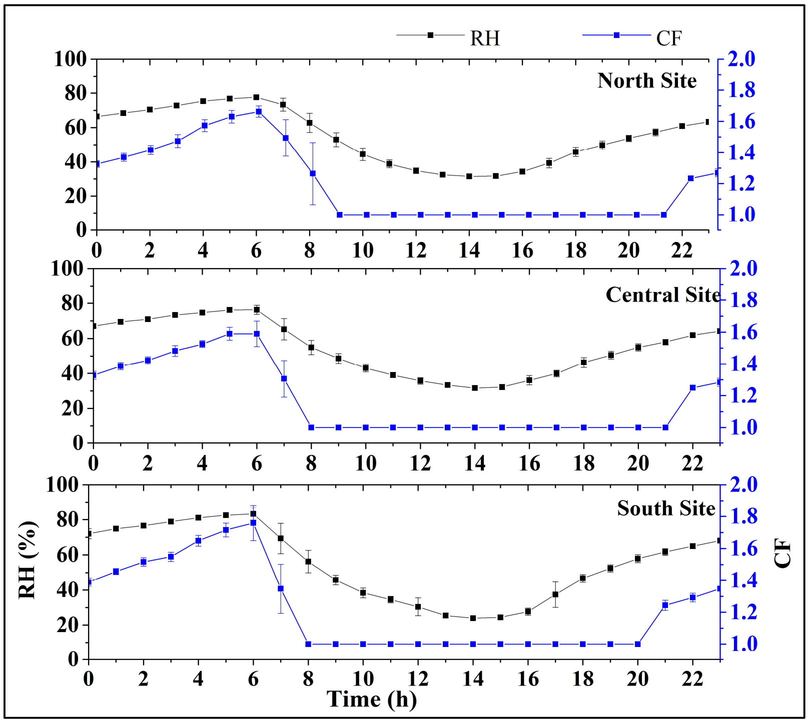

Figure SI.2.1. Median relative humidity (RH) and nephelometer correction factor by time of day and site (see equations 1 and 2 in main text). Error bars represent interquartile ranges (IQR).

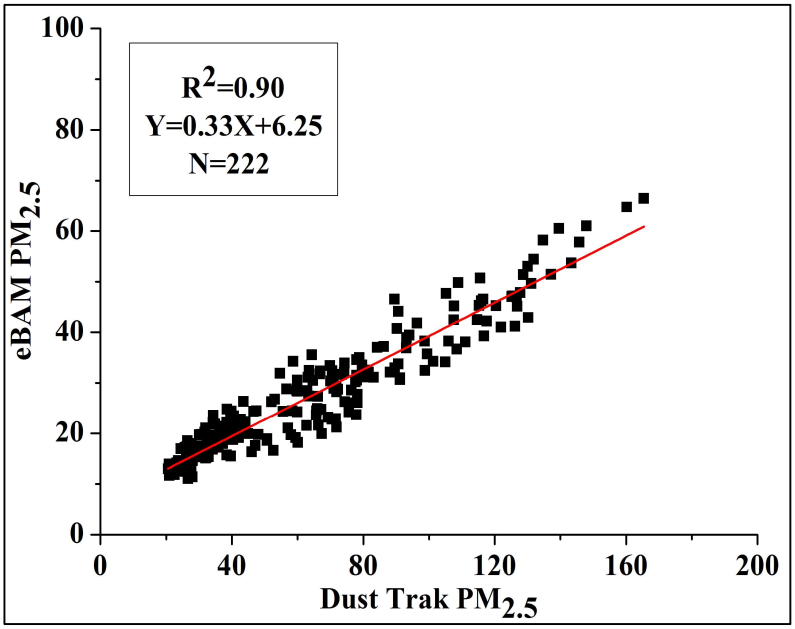

FigureSI.2.2.Comparison between 24h average $PM_{2.5}$ concentrations for collocated eBAM and RH-corrected DustTrak measurements. The best-fit line is the correction factor applied to all DustTrak measurements presented in this study.

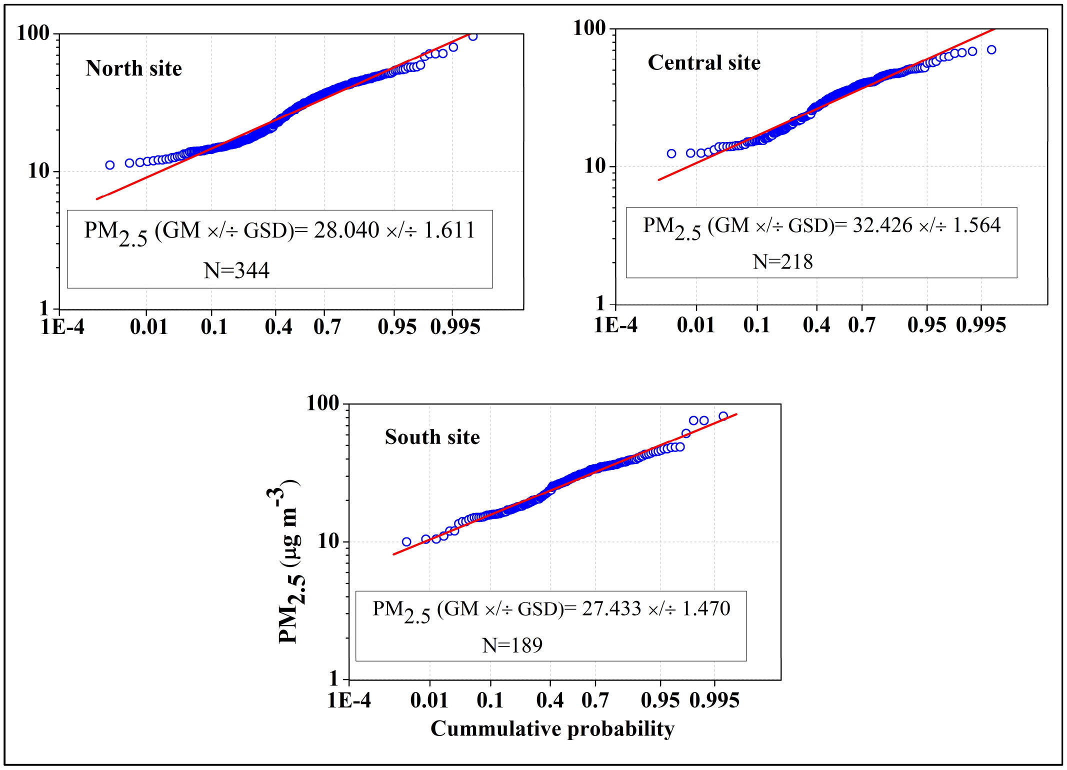

Figure SI.2.3. Lognormal probability plots for 24 h averaged $PM_{2.5}$ concentrations measured at north, central and south sites. The lines within figures indicate best-fit lognormal distributions. Text boxes report geometric mean ×/÷ geometric standard deviation; N represents the number of valid samples for each site.

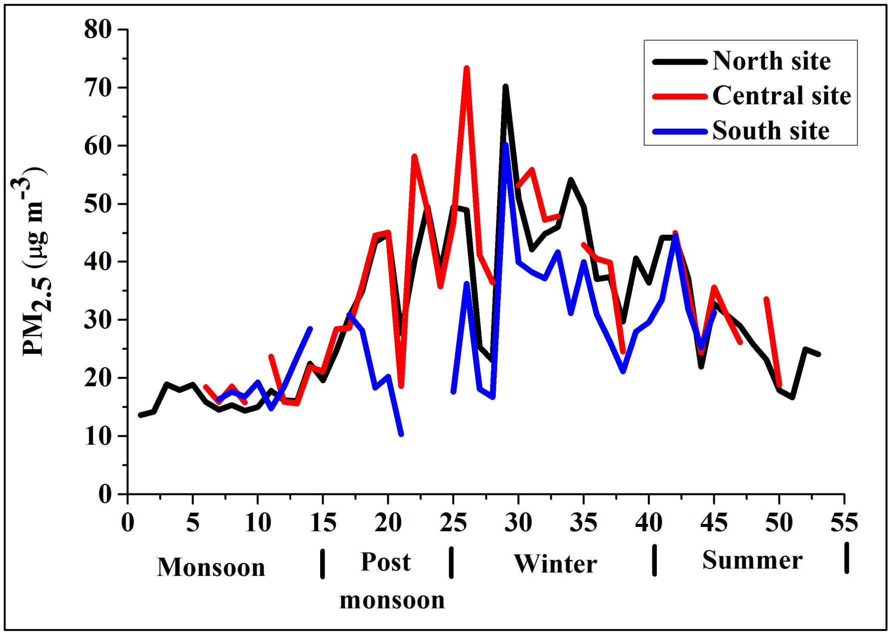

Figure SI. 2.4. Weekly average $PM_{2.5}$concentrations at the three rural ambient sites. Here, week 1 starts11 June 2016.

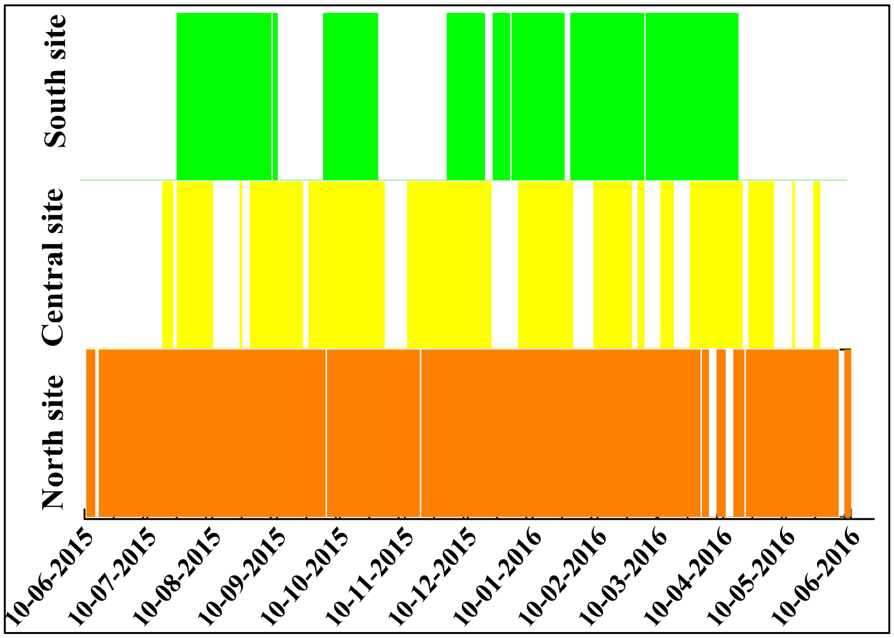

Figure SI.2.5.$PM_{2.5}$ data collection over time(DustTrak and eBAM data) according to site.Gaps represent missing data (e.g., instrument malfunction,power outage). Total data collection: 344 days North site; 218 days Central site; 189 days South site.

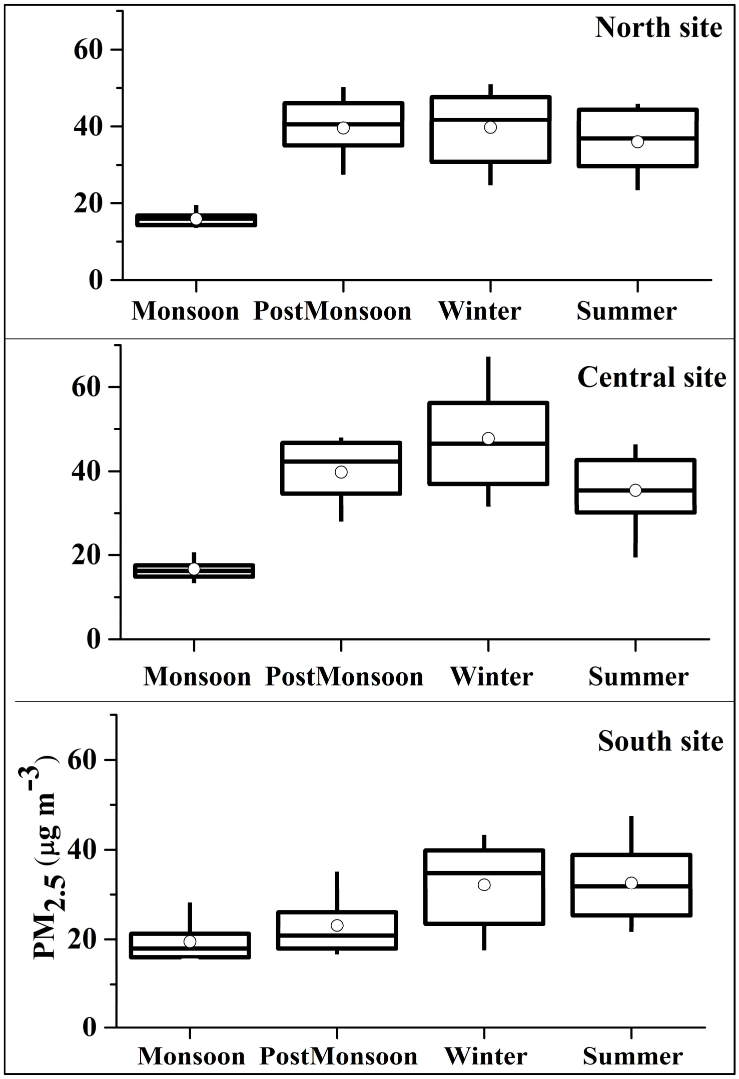

Figure SI.2.6. Variability of 24h averaged $PM_{2.5}$ concentrations according to site and season based on 139 days common data.

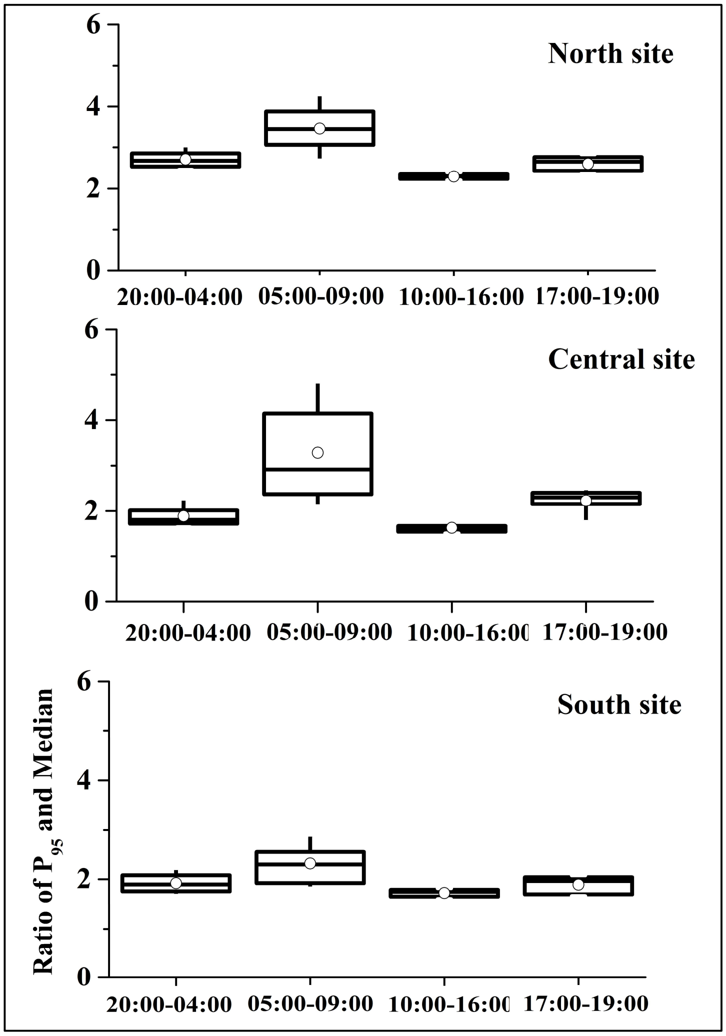

Figure SI. 2.7. Box plots of $PM_{2.5}$ concentrations sampled at the three ambient sites showing a measure of skew (ratio of minute 95th percentile [P95] to 24h median concentration based on 1 min medians of concentration)

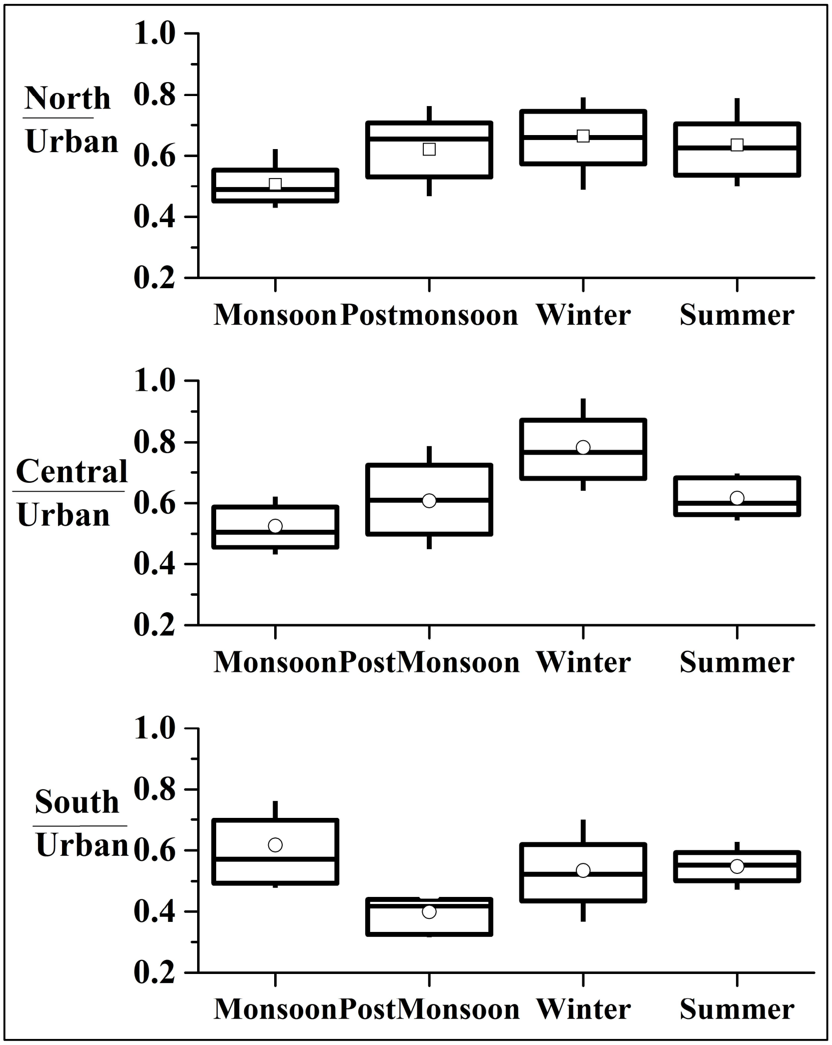

Figure SI.2.8. Season wise distribution of rural-urban $PM_{2.5}$ ratios for three rural sites

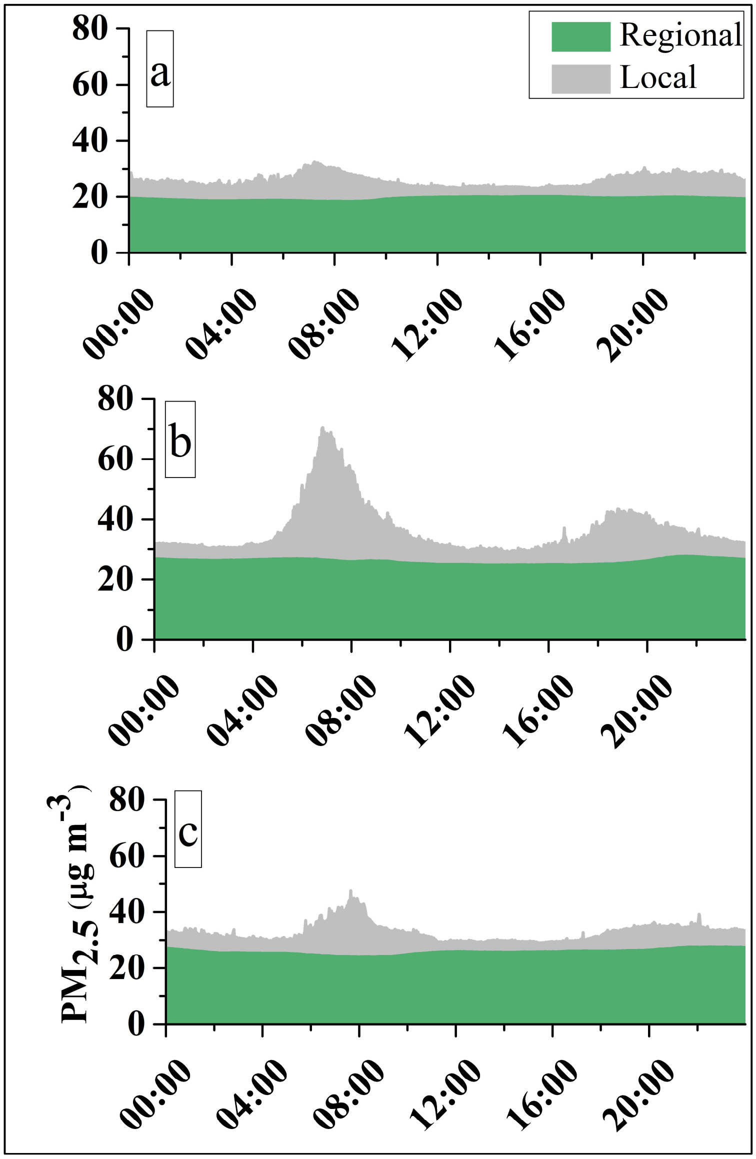

Figure SI.2.9. Median $PM_{2.5}$ concentration by local and regional scale contributions, by time of day at (a) North site (b) Central site and (c) South site location using alternate underwriting function

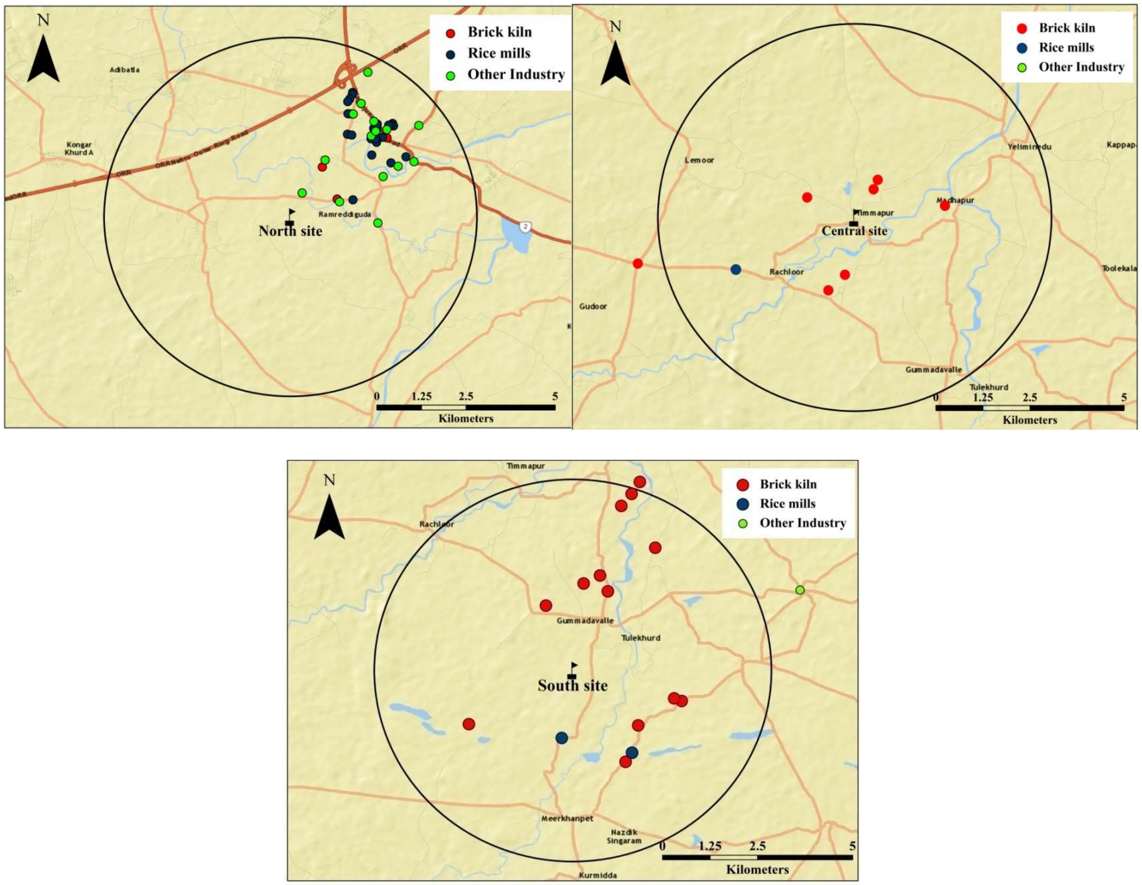

Figure SI.2.10. Aerial view of brick kilns, rice mills and other industries present in 5 km radius around three rural sites.

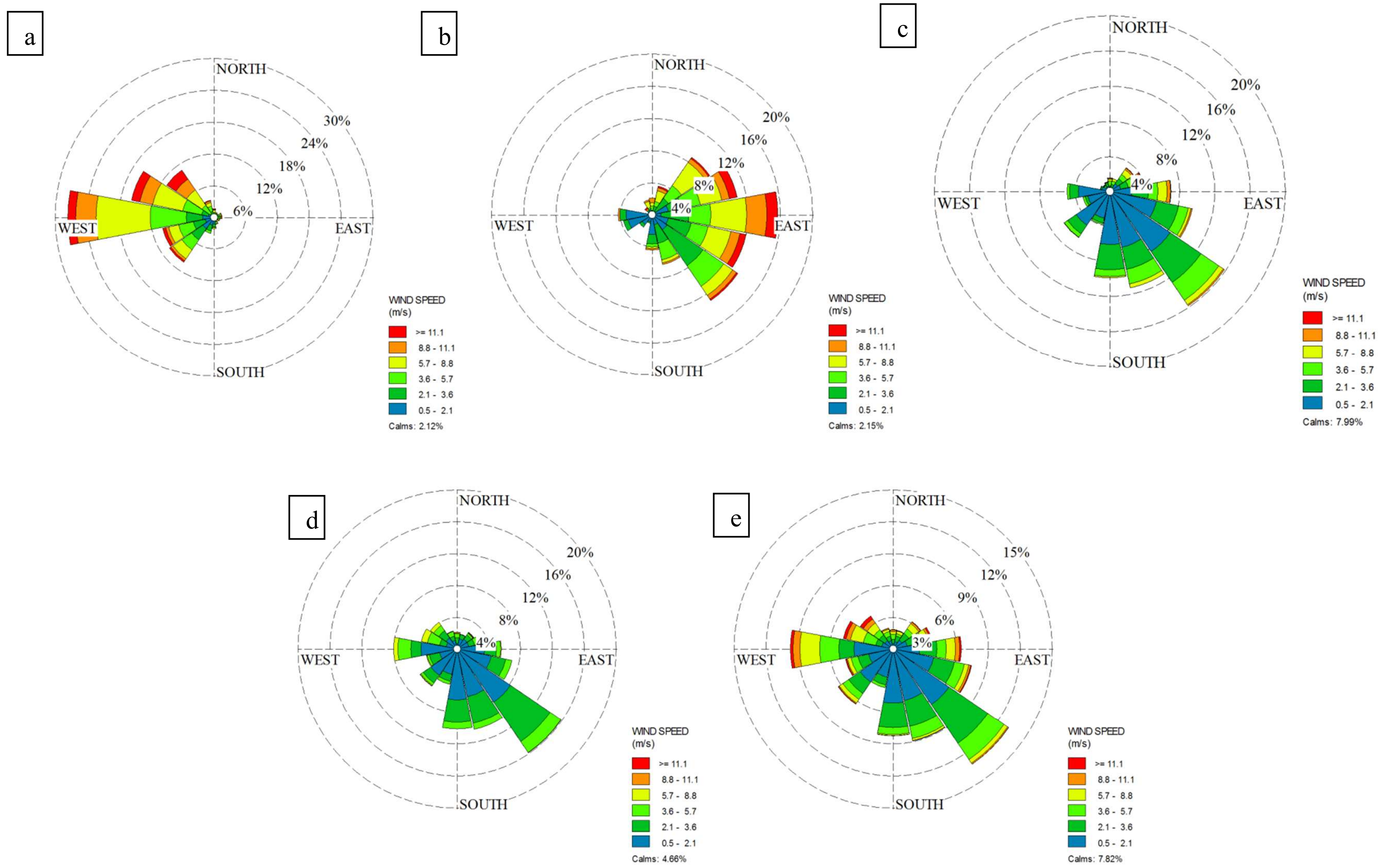

Figure SI.2.11. Wind roses showing wind speed and direction for the (a) monsoon, (b) post monsoon, (c) winter, (d) summer and (e) total monitoring period at the North site. Here and elsewhere, wind rose represent the direction from which wind blows

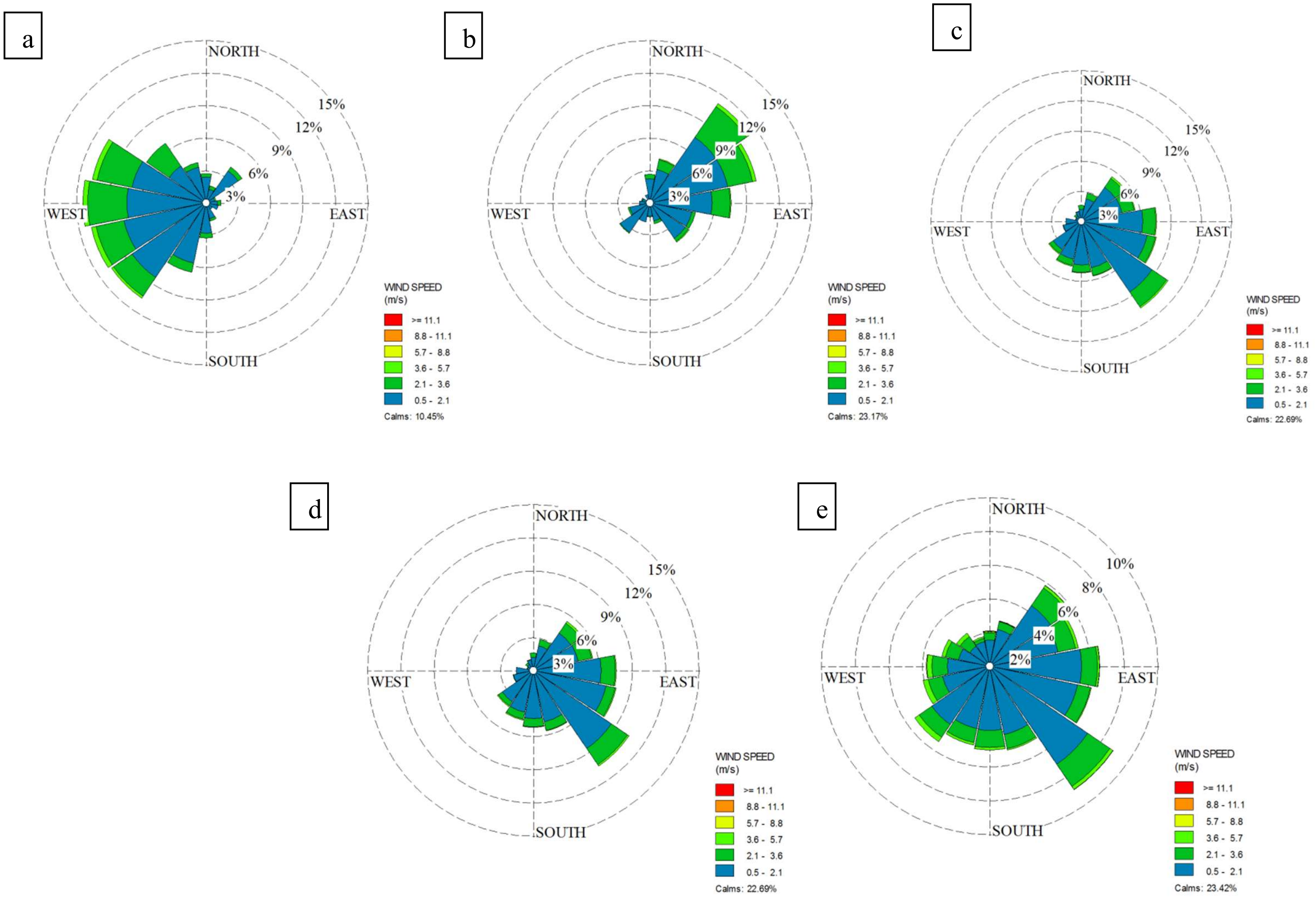

Figure SI.2.12. Wind roses showing wind speed and direction for the (a) monsoon, (b) post monsoon, (c) winter, (d) summer and (e) total monitoring period at Central site.

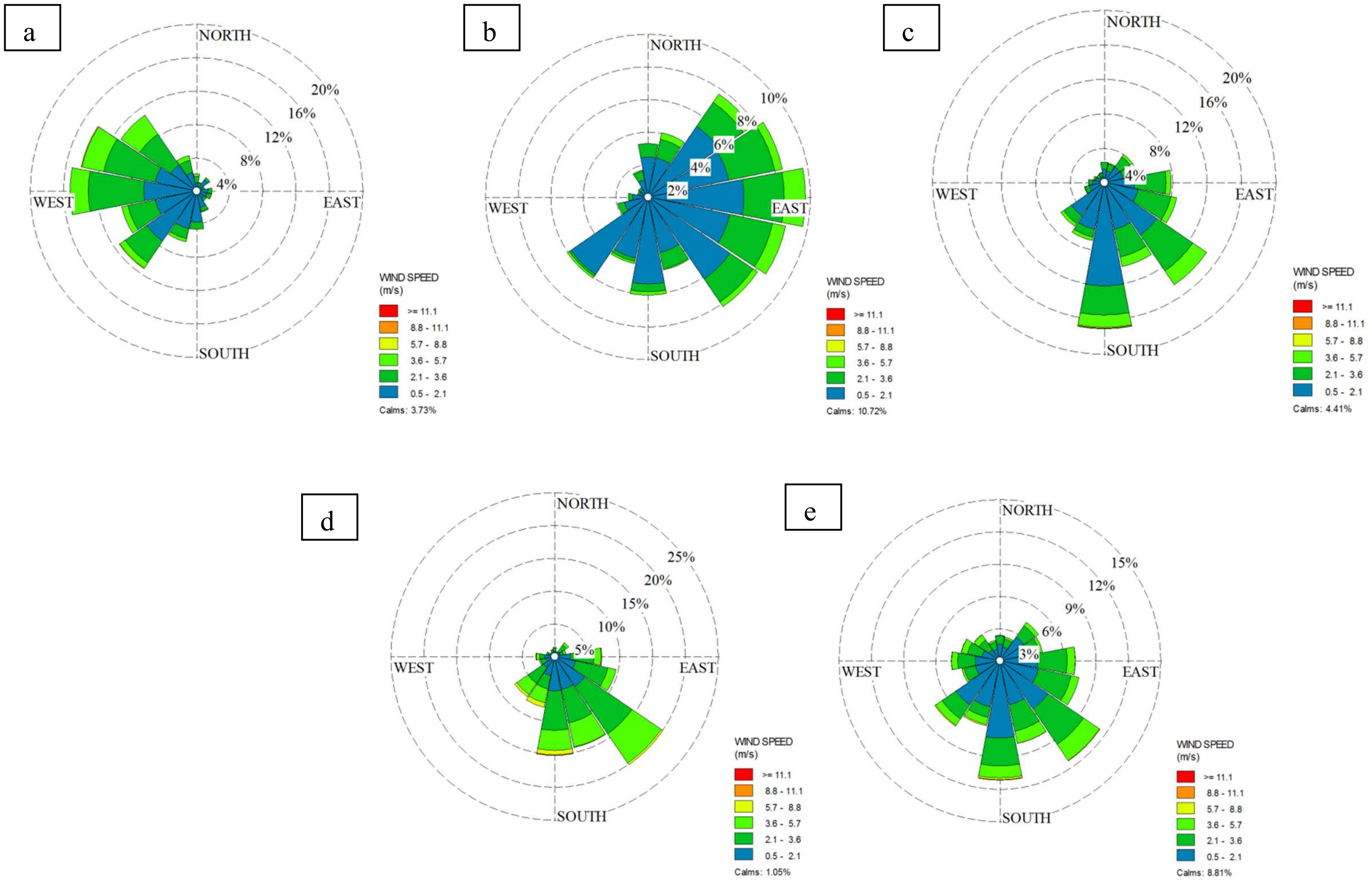

Figure SI.2.13. Wind roses showing wind speed and direction for the (a) monsoon, (b) post monsoon, (c) winter, (d) summer and (e) total monitoring period at South site

Table SI.2.1. $PM_{2.5}$ concentrations (μgm^{-3}) during weekdays and weekends

	North site	Central site	South site
**WeekendMedian (P25, P75)**	29 (18,41)	35 (22,45)	29 (19,37)
**WeekdayMedian (P25, P75)**	29 (19,42)	37 (23,48)	30 (21,36)
**Weekend (24 hr GM ×/÷GSD)**	28.723×/÷ 1.626	32.370×/÷1.576	28.506×/÷1.481
**Weekday (24 hr GM ×/÷GSD)**	27.768×/÷1.606	32.450×/÷1.561	27.036×/÷1.467
**All daysMedian (P25, P75)**	29 (18,41)	35 (23,46)	29 (20,37)
**All days (24 hr GM ×/÷GSD)**	28.040×/÷1.611	32.426×/÷1.564	27.433×/÷1.470

Table SI.2.2 Season wise ratios of medians of 24h averaged $PM_{2.5}$ levels at the three ambient sites

Ratios	$\frac{\text{Central}}{\text{North}}$	$\frac{\text{Central}}{\text{South}}$	$\frac{\text{North}}{\text{South}}$
**Monsoon**	1.03	0.92	0.84
**Post-Monsoon**	1.01	2.21	2.1
**Winter**	1.19	1.47	1.21
**Summer**	0.99	1.08	1.13
**All days**	1.06	1.23	1.15

Table SI. 2.3. Percentage of local concentrations computed based on moving average subtraction method at the different time intervals of a day.

Site	Averagecontributions at different time scales					
	(00:00-23:00)	(00:00-05:00)	(05:00-09:00)	(09:00-17:00)	(17:00-20:00)	(20:00-23:00
**North**	11	12	17	6	9	14
**Central**	12	6	25	8	17	9
**South**	8	7	16	5	7	7

Table SI.2.4. Percentage of local concentrations computed based on alternate underwriting function at the different time intervals of a day

Site	Average contributions at different time scales					
	(00:00-23:00)	(00:00-04:00)	(05:00-08:00)	(09:00-16:00)	(17:00-19:00)	(20:00-23:00
**North**	25	25	35	17	24	30
**Central**	26	15	47	21	34	23
**South**	20	18	33	16	20	21

Table SI. 2.5. Percentage of days with stagnation, recirculation, ventilation, and stagnation-recirculation eventsat three rural sites

Site	Percentage of stagnation days	Percentage of recirculation days	Percentage of Ventilation days	Percentage of days with both stagnation and recirculation events
**North site**	64	44	16	29
**Central site**	63	41	8	34
**South site**	61	49	6	29

Table SI. 2.6. Percentage of stagnation, recirculation and ventilation events during monsoon, post monsoon, winter and summer events at three rural sites.

Site	Event	Monsoon	Post Monsoon	Winter	Summer	Annual
**North site**	Stagnation	2	2	55	41	64
	Recirculation	15	6	42	36	44
	Ventilation	51	15	22	12	16
**Central site**	Stagnation	10	39	39	12	63
	Recirculation	14	26	28	32	41
	Ventilation	55	5	20	20	8
**South site**	Stagnation	18	44	35	3	61
	Recirculation	14	33	36	18	49
	Ventilation	50	7	14	29	6

Table SI. 2.7. Hourly median $PM_{2.5}$ concentrations (µg m^{-3}) during wind speeds. The number of hours of $PM_{2.5}$ episode occurrences is within brackets

Wind speed (m/sec)	North site	Central site	South site
**≤0.5 (calm conditions)**	47 (156)	43(355)	34 (55)
**0.6-2**	38 (222)	34(104)	31 (73)
**2-4**	29 (83)	30(17)	29 (28)
**≥4**	22(49)	23(0)	29 (4)

Table SI.2.8. Hourly median $PM_{2.5}$ concentrations (µg m^{-3}) during various wind direction periods at the three rural sites

Wind direction	North site	Central site	South site
**0≤θ<45**	32	41	37
**45≤θ<90**	37	40	34
**90≤θ<135**	35	38	31
**135≤θ<180**	34	36	30
**180≤θ<225**	32	40	31
**225≤θ<270**	30	37	29
**270≤θ<315**	29	36	27
**315≤θ<360**	27	40	31

Table SI.2.9. Average and standard deviations of monthly temperature and relative humidity (RH) over the study area

	**Temp (°C)**		**RH (%)**	
	**Average**	**SD**	**Average**	**SD**
**Jan**	24.02	6.34	49.71	21.79
**Feb**	27.83	6.4	44.71	22.12
**Mar**	30.85	6.58	39.55	22.69
**Apr**	33.66	6.53	33.42	21.86
**May**	33.19	5.34	42.68	16.65
**Jun**	30.54	5.19	53.89	16.92
**Jul**	29.68	4.41	55.37	15.43
**Aug**	27.49	4.15	68.13	16.08
**Sep**	27.28	4.87	71.34	18.24
**Oct**	27.19	5.45	63.46	20.91
**Nov**	24.8	4.93	61.75	19.4
**Dec**	24.32	5.99	55.16	21.71
